# Neddylation of sterol regulatory element-binding protein 1c is a potential therapeutic target for nonalcoholic fatty liver treatment

**DOI:** 10.1038/s41419-020-2472-6

**Published:** 2020-04-24

**Authors:** Uk-Il Ju, Do-Won Jeong, Jieun Seo, Jun Bum Park, Jong-Wan Park, Kyung-Suk Suh, Jae Bum Kim, Yang-Sook Chun

**Affiliations:** 1 Department of Biomedical Sciences, Seoul National University College of Medicine, Seoul, Korea; 2 Ischemic/Hypoxic Disease Institute, Seoul National University College of Medicine, Seoul, Korea; 3 Department of Hepatobiliary and Pancreatic Surgery, Seoul National University Hospital, Seoul, Korea; 4 National Creative Research Initiatives Center for Adipose Tissue Remodeling, Institute of Molecular Biology and Genetics, Department of Biological Sciences, Seoul National University, Seoul, Korea; 5 Department of Physiology, Seoul National University College of Medicine, Seoul, Korea

**Keywords:** Neddylation, Neddylation, Metabolic syndrome, Metabolic syndrome

## Abstract

Nonalcoholic fatty liver disease (NAFLD) is a risk factor for progression of steatohepatitis, liver cirrhosis, and liver cancer. Although pathological condition of NAFLD, which arises from an excessive accumulation of triglyceride in the liver, is accompanied by elevated sterol regulatory element-binding protein 1c (SREBP1c) level, it is largely unknown which factors are involved in the modification of SREBP1c. In this study, we discovered that neddylation of SREBP1c competes with its ubiquitination and stabilizes SREBP1c protein level, and eventually promotes hepatic steatosis. We also demonstrated that human homolog of mouse double minute 2 (HDM2) acts as an E3 neddylation ligase of SREBP1c. Further, treatment with the neddylation inhibitor, MLN4924, attenuates high-fat diet-induced hepatic steatosis by reducing the levels of SREBP1c protein and hepatic triglyceride. Our results indicate that the blockade of SREBP1c neddylation could be a novel approach in the defense against NAFLD.

## Introduction

Nonalcoholic fatty liver disease (NAFLD) is a common disease in both developed and developing countries, and is a precursor of the more advanced liver diseases including nonalcoholic steatohepatitis, liver cirrhosis, and liver cancer^1,2^; therefore, its prevention is an important clinical goal. NAFLD is characterized by the accumulation of excess liver triacylglycerol (TG) and of the TG accounted for in liver, 26% arise from de novo synthesis^3^. In addition, patients with NAFLD have threefold higher rates of lipogenesis than healthy individuals^4^, suggesting that control of lipogenic pathway can be the potential target to NAFLD. Currently, the increasing incidence of obesity is recognized as a major etiological factor for hepatic steatosis; therefore, exercise, weight loss and dietary control are well accepted as the treatments for hepatic steatosis^5,6^. In pharmacological therapy, ursodeoxycholic acid (UDCA) is the only therapeutic agent used to treat hepatic steatosis^7^. However, its clinical outcome remains controversial^8^. Insulin sensitizers and hepatic protective agents have been also examined in the treatment of NAFLD, but significant clinical effects were not successfully demonstrated, indicating the lack of effective drugs specifically targeting NAFLD^9,10^.

Sterol regulatory element-binding protein-1c (SREBP1c) is a critical regulator governing lipid homeostasis in liver^11,12^. Chronic activation of SREBP1c increases lipogenic activity and contributes to the progression of hepatic steatosis, which can then develop to cirrhosis and liver failure^13^. In in vivo and in vitro conditions, increased rates of fatty acid synthesis by SREBP1c contribute to the development of hepatic steatosis in obese mice^14^. Inhibition of SREBP1c expression in the liver of obese mice results in 50% reduction of hepatic TG^15^. For these reasons, SREBP1c has been focused as a target for the treatment of hepatic steatosis. Despite importance of SREBP1c in NAFLD, most studies have only concentrated on inhibiting the transcriptional activity of SREBP1c, and post-translational modifications of SREBP1c remains unclear.

The neural precursor cell-expressed, developmentally downregulated 8 (NEDD8) is ubiquitously expressed in mammalian tissues and is structurally similar to ubiquitin^16^. Neddylation is the process by which NEDD8 is covalently conjugated to lysine residues of the substrates by sequential enzymatic reaction of neddylation-activating enzyme, a conjugating enzyme and E3 ligase^17,18^. Unlike ubiquitination that mainly degrades proteins, neddylation has diverse roles including regulation of protein activity^19,20^ and the stability of its substrates^21^.

MLN4924 is a first-in-class highly selective NEDD8-activating enzyme (NAE) inhibitor and has been evaluated in phase I/II clinical trials^22,23^. As demonstrated in several studies, MLN4924 exhibits antitumor activities by activating NF-kB and mammalian target of rapamycin (mTOR)^24,25^. In addition, MLN4924 was found to induce p21 and p27 accumulation in a variety of solid and hematologic malignancies^26–28^. Recently, emerging evidence has been suggested that neddylation plays a role as an important regulator of lipid synthesis. A DNA microarray study in ob/ob mice demonstrated that expression of NEDD8 was upregulated, whereas ubiquitin was downregulated^29^. Moreover, neddylation is also known to stimulate adipocyte differentiation and fat accumulation in adipocytes^30^. Also, NEDD8-targeting siRNAs or MLN4924 was found to effectively prevent high-fat-diet-induced obesity and glucose intolerance in mice^21^. Although antiobesity effect of MLN4924 was evaluated, its contribution to the physiopathology of hepatic steatosis and related molecular mechanism is still unclear.

Herein, we demonstrated a role for human homolog of mouse double minute 2 (HDM2)-mediated neddylation of SREBP-1c in the progression of hepatic steatosis. NEDD8 is highly expressed in human fatty livers and positively correlates with SREBP1c. Moreover, by interfering with ubiquitination, HDM2-mediated neddylation stabilizes SREBP-1c protein level and consequently drives lipid accumulation in the liver. Thus, the inhibition of SREBP1c neddylation could be a potential strategy for treating fatty liver.

## Results

### Expression of NEDD8 is positively associated with increased lipogenesis transcription factors and is involved in the development of hepatic steatosis

**Fig. 1:**
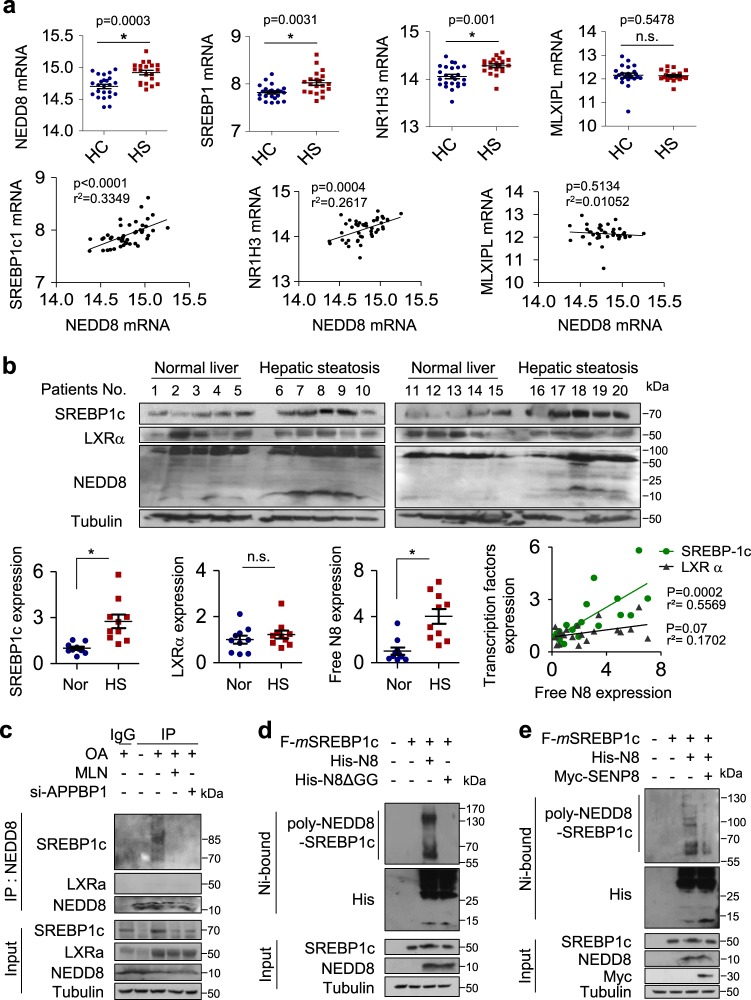
Expression of NEDD8 is positively associated with increased lipogenesis transcription factors and is involved in the development of hepatic steatosis. **a** Dot plot of NEDD8 expression levels (ILMN_2058070 at probe), SREBP1c (ILMN_1695378 at probe), NR1H3 for LXRα (ILMN_1695378 at probe), and MLXIPL for ChREBP (ILMN_1722073 at probe) within HC and HS patients (upper). Correlation analysis of NEDD8 and SREBP1c, NR1H3, and MLXIPL expression in the livers of HC and HS patients (bottom panel). The *y*-axis represents log2 expression of genes and error bars show SEM. **b** Normal liver tissue and hepatic steatosis tissue were subjected to immunoblotting using antibodies to NEDD8, SREBP1c, and LXRα. Protein band intensity was analyzed using ImageJ and protein levels were normalized relative to β-tubulin. Normal liver (blue symbols), hepatic steatosis (red symbols); correlation analysis was then performed between NEDD8 and SREBP1c or LXRα (green symbols: SREBP1c vs. NEDD8 *r*^2^ = 0.8588, black symbols: LXRα vs. NEDD8 *r*^2^ = 0.7038). *P < 0.0001 (*n* = 10 per group). **c** SREBP1c–NEDD8 interactions were examined by immunoprecipitation experiments in HepG2 cells treated with OA (500 μM) for 24 h. **d** HEK293 cells were cotransfected with Flag-SREBP1c and His-NEDD8 or His-NEDD8ΔGG for 48 h and subjected to Ni^2+^ pull-down assay. Purified cells were analyzed by Western blotting. **e** Flag-SREBP1c plasmid was cotransfected with His-NEDD8 or Myc-SENP8 into HEK293 cells for 48 h, and proteins isolated using Ni^2+^ pull-down assay were analyzed by Western blotting.

To evaluate the potential importance of neddylation in the pathogenesis of NAFLD, we first analyzed mRNA expressions of NEDD8 and lipogenic transcription factors using the omnibus dataset (GSE89632). The expression levels of *NEDD8* mRNA are higher in liver tissues of hepatic steatosis patients than those of healthy subjects. Among lipogenesis-related transcription factors, SREBP1 and NR1H3 (LXR) genes were elevated in liver tissues of hepatic steatosis patients, but MLXIPL (ChREBP) genes remained unchanged (Fig. 1a, upper panel). In addition, the increased expression of SREBP1 and NR1H3 positively correlated with NEDD8 expression (Fig. 1a, bottom panel). To further examine whether NEDD8 is involved in hepatic steatosis, we analyzed NEDD8, SREBP1c, and LXRα protein levels in the liver tissues from hepatic steatosis patients and normal subjects. When the proteins were normalized to β-tubulin, NEDD8 and SREBP1c were upregulated in steatotic livers. LXRα appeared to be induced slightly, but not significantly on statistics. NEDD8 showed a positive correlation with either SREBP1c or LXRα, of which SREBP1c is associated with NEDD8 more strongly (Fig. 1b). Given the omnibus dataset and clinical data (Fig. 1a, b), we next performed immunoprecipitation (IP) experiments to determine which transcription factors interacted with NEDD8. As oleic acid (OA) has been reported to induce hepatic steatosis^31^, OA was treated for 24 h to make similar condition of NAFLD in HepG2 cells. As shown in Fig. 1c, IP assays revealed that NEDD8 only bound to SREBP1c, and not to LXRα. Interestingly, the SREBP1c protein, which was coprecipitated with NEDD8, was shown to be roughly 85 kDa on PAGE. Considering the molecular weight of naïve SREBP1c (~70 kDa), the covalent bonding of small peptide(s) could make an electrophoretic mobility shift of SREBP1c, which preliminarily suggests the NEDDylation of SREBP1c. The interaction between SREBP1c and NEDD8 was abolished by an inhibitor MLN4924 or an siRNA for the E1 enzyme APPBP1, indicating that SREBP1c is NEDDylated (Fig. 1c). To identify the neddylated SREBP1c, we performed a Ni^2+^ pull-down assay. This showed that SREBP1c is covalently bonded by His-NEDD8 but not by His-NEDD8ΔGG (a conjugation-defective Gly-75/76 deletion mutant) (Fig. 1d). In addition, SREBP1c was deneddylated by SENP8, which is known to remove NEDD8 from neddylated proteins (Fig. 1e). Taken together, these results suggest that high expression of NEDD8 is associated with increased lipogenic transcription factors and among them, only SREBP1c was covalently conjugated with NEDD8.

### The neddylation upregulates SREBP1c transcriptional activity and protein level

**Fig. 2:**
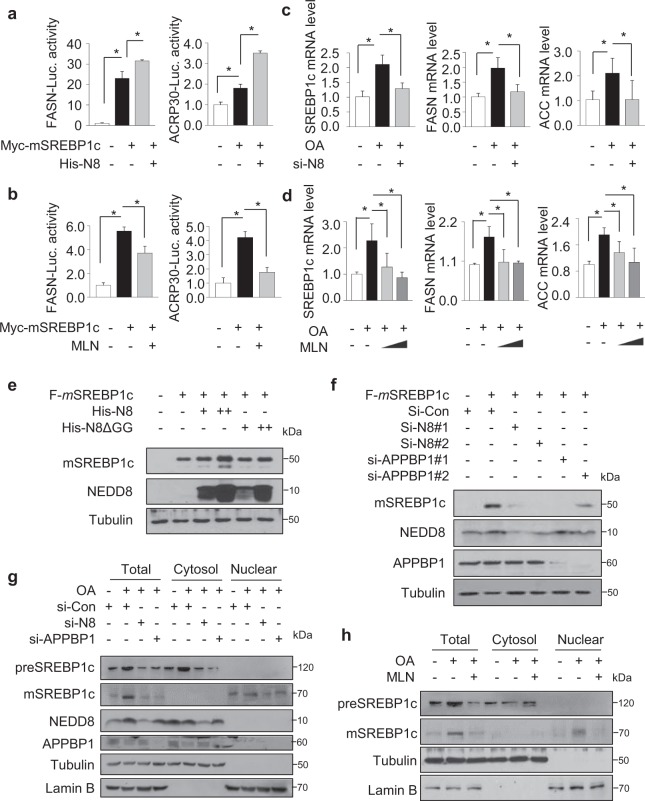
The neddylation upregulates SREBP1c transcriptional activity and protein level. **a** HEK293 cells were cotransfected with luciferase plasmid and the other plasmids listed, incubated for 48 h, and subjected to luciferase assay normalized relative to β-galactosidase activity. Data are expressed as means ± SD (*n* = 3). *P < 0.05 versus control group. **b** Transfected HepG2 cells were treated with MLN4924 (500 nM) and incubated for 24 h; luciferase activity was then measured. Results were normalized relative to β-galactosidase activity. **c** HepG2 cells were transfected with 50 nM siRNA for NEDD8 and treated with 500 μM OA for 18 h. RT-qPCR was performed to evaluate the expression of lipogenic genes. The mRNAs were quantified in reference to the 18S RNA levels, and presented as the means ± SD (*n* = 3). * denotes *P* < 0.05 versus the control group. **d** HepG2 cells were incubated with 500 μM OA and 250/500 nM MLN4924 for 18 h. Cells were subjected to RT-qPCR. **e** HepG2 cells were cotransfected with the Flag-SREBP1c and His-NEDD8 or His-NEDD8ΔGG plasmids for 48 h. Cell lysates were subjected to Western blotting to verify protein expression. **f** HepG2 cells were cotransfected with the indicated siRNAs (50 nM each) and Flag SREBP1c plasmid for 48 h. Proteins were analyzed by Western blotting. **g** HepG2 cells were transfected with the indicated siRNAs (50 nM each) for 24 h, and cells were treated with OA (500 μM) for a further 24 h. Nuclear extraction was performed in the cell lysates. Extraction samples were analyzed by Western blotting. **h** HepG2 cells were incubated with OA (500 μM), and 24 h later treated with MLN4924 for a further 24 h. Nuclear extraction was performed, and samples analyzed by immunoblotting.

Given that, among the lipogenic transcription factors, SREBP1c was only conjugated with NEDD8 (Fig. 1c−e), we next investigated the effects of neddylation on transcriptional activity of SREBP1c. Overexpression of SREBP1c transactivated the promoters of FASN and adiponectin (Acrp30) genes and coexpression with His-NEDD8 further enhanced its transcriptional activity (Fig. 2a). However, MLN4924 suppressed the transcriptional activity of SREBP1c (Fig. 2b). Next, we treated cells with OA to stimulate the induction of SREBP1c and its target genes (FASN and ACC)^32^. As expected, the mRNAs of SREBP1c, FASN and ACC were increased by OA. However, such an effect of OA was markedly diminished by MLN4924 and NEDD8 knockdown (Fig. 2c, d). Further, we examined the effects of neddylation on SREBP1c protein level. Ectopically expressed NEDD8 clearly increased SREBP1c protein level, but NEDD8ΔGG did not (Fig. 2e). Conversely, knockdown of NEDD8 or APPBP1 decreased ectopically expressed SREBP1c (Fig. 2f). Also, the OA-induced expressions of SREBP1c precursor and mature proteins were attenuated by si-NEDD8, si-APPBP1 or MLN4924 (Fig. 2g, h). Altogether, these results suggest that neddylation is essential for both transcriptional activity and protein expression of SREBP-1c.

### Neddylation stabilizes SREBP1c protein by competitively interfering with ubiquitination

**Fig. 3:**
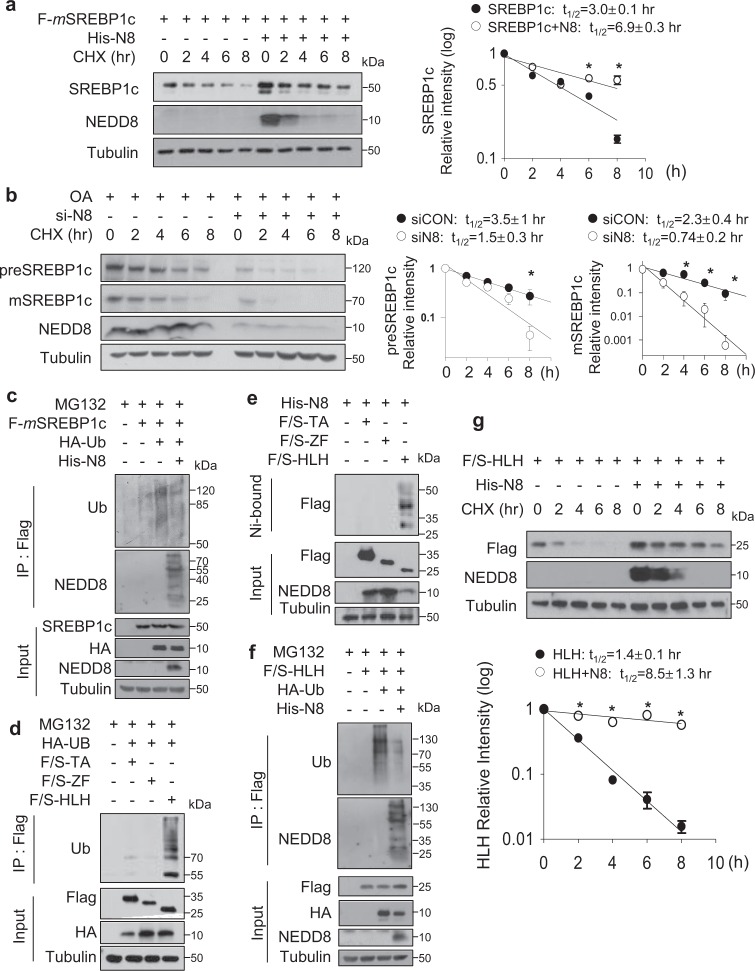
Neddylation stabilizes SREBP1c protein by competitively interfering with ubiquitination. **a** HEK293 cells, which had been cotransfected with Flag-SREBP1c and His-NEDD8 for 48 h, were treated with cycloheximide for the indicated time, and then cell lysates were subjected to Western blotting. Band intensities (mean ± SD, *n* = 3) on blots were analyzed using ImageJ and plotted. *P < 0.05 versus control group. **b** HepG2 cells, which were transfected with NEDD8, were treated with OA. After incubated for 24 h, cells were treated with cycloheximide for the indicated time and then subjected to Western blotting. Protein band intensity was analyzed using the ImageJ software. * denotes *P* < 0.05 versus the control. **c** HEK293 cells were cotransfected with Flag-SREBP1c, His-NEDD8 and HA-Ubiquitin for 24 h and treated with MG132 (10 μM) for 8 h. After the treatment, immunoprecipitation was performed using anti-Flag beads and assessed by Western blotting. **d** HEK293 cells were cotransfected with indicated plasmids for 24 h. After transfection, 10 μM of MG132 was added to the cells. Cells lysates were purified with anti-Flag beads, and proteins were analyzed by Western blotting. **e** The SREBP1c domains and His-NEDD8 plasmids were cotransfected into HEK293 cells. Transfected cells were subjected to Ni^2+^ pull-down assay, and immunoblotted using anti-Flag antibody. **f** HEK293 cells were cotransfected with the indicated plasmids for 24 h. After transfection, MG132 was added to the cells at 10 μM. Cell lysates were purified with anti-Flag beads, and proteins were analyzed by Western blotting. **g** Transfected HEK293 cells were treated with cycloheximide for the indicated times, and cell lysates were subjected to immunoblotting. Band intensities (mean ± SD, *n* = 3) on blots were analyzed using ImageJ and plotted. *P < 0.05 compared to control group.

To evaluate the molecular mechanism underlying neddylation-mediated SREBP1c protein increase, we examined the stability of SREBP1c protein in the presence of cycloheximide, an inhibitor of protein synthesis. The ectopic expression of NEDD8 markedly increased the half-life of SREBP1c (Fig. 3a). Conversely, SREBP1c precursor and mature proteins were destabilized by NEDD8 knockdown (Fig. 3b). Given that our previous research suggested neddylation stabilizes PPARγ by competing with ubiquitination^21^, we next examined the possibility that neddylation competes with SREBP1c ubiquitination. Indeed, ubiquitination of SREBP1c was largely reduced by ectopic expression of NEDD8 (Fig. 3c). As ubiquitination of SREBP1c is known to occur in the helix–loop–helix (HLH) domain^33^, three segments of SREBP1c (TA, transactivation; ZF, Zinc-finger; HLH) were generated. IP assay revealed ubiquitination occurs only in the HLH domain (Fig. 3d) and HLH domain was also neddylated (Fig. 3e). Because both neddylation and ubiquitination of SREBP1c occur in the same domain, we next confirmed whether neddylation interferes with the ubiquitination in the HLH domain of SREBP1c. As shown as Fig. 3f, the level of ubiquitinated HLH domain was dramatically decreased by ectopic expression of NEDD8. In accordance with these results, the stability of the HLH domain was markedly increased by the ectopic expression of NEDD8 (Fig. 3g). These results suggest that the enhanced SREBP1c expression by neddylation is mediated by competition with ubiquitination.

### HDM2 interacts with SREBP1c and promotes its neddylation

**Fig. 4:**
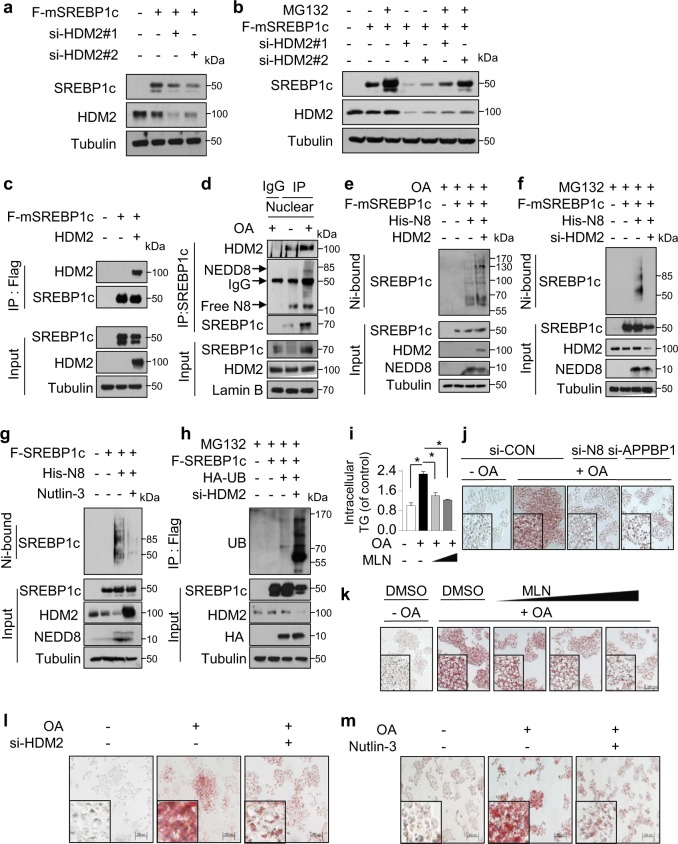
HDM2 interacts with SREBP1c and promotes its neddylation. **a** HepG2 cells were cotransfected with HDM2 siRNAs (50 nM each) and Flag-SREBP1c for 48 h. Cell lysates were analyzed by Western blotting. **b** HepG2 cells were cotransfected with Flag-SREBP1c and/or si-HDM2 (50 nM each) for 48 h, and then treated with 10 μM MG132 for 8 h. Cell lysates were analyzed by immunoblotting. **c** HEK293 cells were cotransfected with Flag-SREBP1c and HDM2 for 48 h, and the cell lysates were assessed by immunoprecipitation using Flag beads. Precipitated proteins were eluted with 1× FLAG peptide and subjected to Western blotting. **d** HepG2 cells were exposed to OA (500 μM) for 24 h. Nuclear extraction was performed in the cell lysates. The nuclear lysates were immunoprecipitated by anti-SREBP1c antibody, and purified proteins were analyzed by immunoblotting. **e** HepG2 cells were cotransfected with the indicated plasmids for 24 h and exposed to OA (500 μM) for 24 h. Cell lysates were subjected to immunoblotting after Ni^2+^ pull-down assay. **f** HepG2 cells were cotransfected with Flag-SREBP1c, His-NEDD8 and HDM2 siRNA (50 nM), and 24 h later were incubated with MG132 (10 μM) for 8 h. Cell lysates were subjected to Ni^2+^ pull-down assay and purified proteins analyzed by Western blotting. **g** Flag-SREBP1c plasmid was cotransfected with His-NEDD8 into HepG2 cells for 24 h and treated with Nutlin-3 (10 μM) for 24 h. Proteins isolated using Ni^2+^ pull-down assay were analyzed by Western blotting. **h** HepG2 cells were cotransfected with indicated siRNA (50 nM) and plasmids for 24 h, and treated with MG132 for 8 h. Cell lysates were subjected to immunoprecipitation by anti-Flag beads, and performed Western blotting. **i** HepG2 cells were treated with OA for 24 h and then treated with MLN4924 (250 nM, 500 nM) for an additional 24 h. Intracellular triglyceride levels were measured by using EnzyChrom™ Triglyceride Assay Kit. Data were expressed as means ± SD of three independent experiments. *P < 0.05 versus control group. **j** HepG2 cells were transfected with the indicated siRNAs (50 nM each), and treated with OA (500 μM) 48 h later for a further 24 h. Cells were subjected to Oil Red O staining to visualize accumulated lipid droplets in the cells. Representative images of Oil Red O staining were captured in HepG2 cells at ×200 magnification. **k** HepG2 cells were treated with OA (500 μM) for 24 h, and treated with MLN4924 (125, 250, or 500 nM) for 24 h. Oil Red O staining was performed on fixed cells to detect lipid droplets. Representative images of Oil Red O staining were captured at ×200 magnification. **l** HepG2 cel**l**s were transfected with the indicated siRNAs (50 nM), and 48 h later treated with OA (500 μM) for a further 24 h. Inhibitory effect of HDM2 knockdown on lipid accumulation was confirmed through Oil Red O staining. **m** HepG2 cells were incubated with OA (500 μM) for 24 h, and treated with Nutlin-3 (10 μM) for an additional 24 h. Inhibitory effect of Nutlin-3 on lipid accumulation was confirmed through Oil Red O staining.

Based on the above results, we next examined several E3 ligases to determine which E3 ligase is involved in SREBP1c neddylation. The results indicated that HDM2, known as E3 ligase for PPARγ^21^, acts as an E3 ligase of SREBP1c neddylation (data not shown). In agreement with this result, knockdown of HDM2 by siRNAs decreased SREBP1c expression (Fig. 4a), which was restored by MG132, a proteasomal inhibitor (Fig. 4b). To confirm whether HDM2 could interact with SREBP1c, we performed IP analysis after transfection of HDM2 and SREBP1c. Ectopically expressed HDM2 physically interacted with SREBP1c (Fig. 4c). In addition, endogenous SREBP1c interacted with both HDM2 and NEDD8, and the NEDD8 immunoblotting showed that a bigger form (~85 kDa) of SREBP1c is conjugated with NEDD8 (Fig. 4d). Next, to confirm whether HDM2 is indeed responsible for SREBP1c neddylation, we performed Ni^2+^ pull-down assay. As shown in Fig. 4e, HDM2 overexpression significantly increased SREBP1c neddylation. Conversely, SREBP1c neddylation was reduced by HDM2 knockdown using siRNA transfection or Nutlin-3 treatment, an inhibitor of HDM2 (Fig. 4f, g). Moreover, the ubiquitination of SREBP1c was enhanced by knockdown of HDM2 (Fig. 4h). Based on a report that OA induces SREBP1c protein level, TG and lipid accumulation in liver^31^, we next tested whether neddylation could regulate hepatic TG or lipid accumulation. As shown as Fig. 4i, MLN4924 clearly decreased OA-induced TG accumulation. Also, knockdown of NEDD8 and APPBP1 or MLN4924 treatment decreased OA-induced lipid accumulation in HepG2 cells (Fig. 4j, k). Next, we also examined the effect of HDM2 on lipid accumulation. Both HDM2 knockdown and Nutlin-3 treatment significantly reduced OA-induced lipid accumulation (Fig. 4l, m). These results indicate that HDM2 acts as an E3 ligase promoting SREBP1c neddylation and stabilizes SREBP1c protein.

### Neddylation blockade by MLN4924 prevents HFD-induced hepatic steatosis of mice

**Fig. 5:**
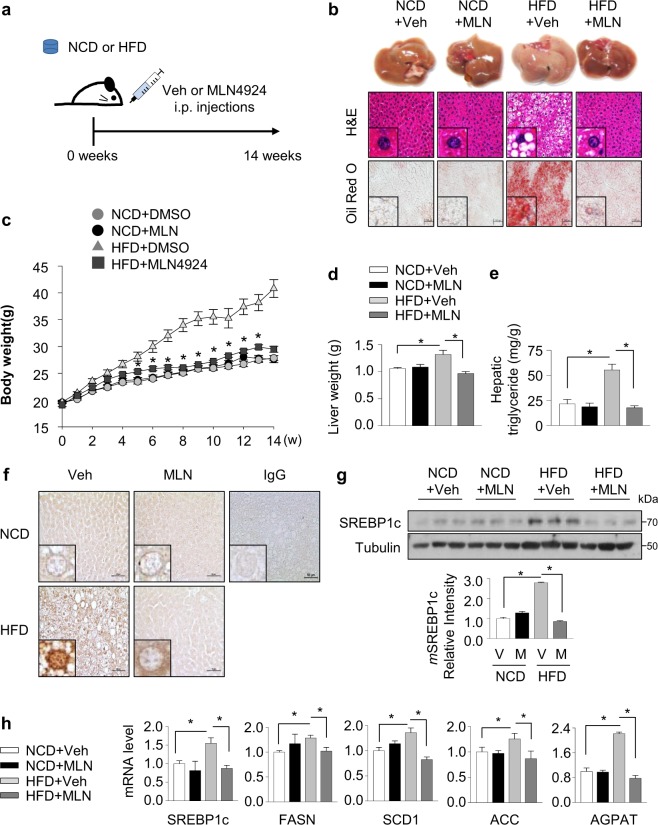
Neddylation blockade by MLN4924 prevents HFD-induced hepatic steatosis of mice. **a**−**e** Mice were divided into four groups (*n* = 9 per group): vehicle treatment of NCD-fed mice or HFD-fed mice, MLN4924 treatment (30 mg/kg) of NCD-fed mice or HFD-fed mice. Mice were fed HFD for 12 weeks, and MLN4924 was injected intraperitoneally twice weekly for 12 weeks. **a** Schematic diagram of in vivo model. **b** Representative images showing the morphology (top), H&E staining (middle) and Oil O Red staining (bottom) of liver sections from HFD-fed mice (*n* = 9 images per group). **c** Mice were weighed for 12 weeks. **d** Liver weight measured after 12 weeks (left). **e** Hepatic triglyceride levels were measured in extracted liver tissues (right). The data are presented as the means ± SD (*n* = 9 per group). *P < 0.05 versus control group. **f** The expression of SREBP1c in liver tissues was analyzed by immunohistochemistry using anti-SREBP1c antibody and IgG used as a negative control (*n* = 9 per group). Representative images of immunohistochemistry were captured in liver tissues with ×400 magnification. **g** Western blots for the expression of SREBP1c in the livers of four groups of mice. The band intensities of SREBP1c protein were calculated using ImageJ and plotted by graph (mean ± SD, *n* = 3). **h** Total RNAs was extracted from liver tissues of four groups of mice. SREBP1c, FASN, SCD1, ACC, and AGPAT mRNA levels were quantified by RT-qPCR. Results were quantified as relative levels vs. 18S RNA level. All data were presented as the mean ± SD (*n* = 9 per group).

To investigate the protective contribution of SREBP1c neddylation to NAFLD progression, mice given a normal chow diet (NCD) or high-fat diet (HFD) were injected with vehicle or MLN4924 for 12 weeks simultaneously (Fig. 5a). The livers of HFD-fed mice were significantly enlarged and pale, showing the typical appearance of a steatotic liver. In contrast, the livers of MLN4924-treated HFD-fed mice were similar in appearance to those of mice given NCD. Similar results were obtained with hematoxylin and eosin (H&E) and Oil Red O staining, indicating that treatment with MLN4924 remarkably reduced hepatic steatosis (Fig. 5b). Moreover, body and liver weights of MLN4924-treated HFD-fed mice showed similar to those of NCD-fed mice, while HFD-fed mice had heavier weights than NCD-fed mice (Fig. 5c, d). Given no significant difference in the amount of food intake between the NCD and HFD groups, mice in two groups seem to have similar appetite (Supplementary Fig. S1A). In addition, the levels of hepatic triglyceride were also decreased in the liver tissues of HFD-fed mice treated with MLN4924 (Fig. 5e). As SREBP1c is known to be highly expressed in hepatic steatosis, we analyzed SREBP1c protein level using immunohistochemistry (IHC) analysis. As shown as Fig. 5f, high expression of SREBP1c was shown in the livers of mice fed HFD for 12 weeks. However, the expression of SREBP1c was remarkably decreased in the livers of MLN4924-treated HFD-fed mice. In further Western blot analysis, the level of SREBP1c was coincident with IHC results (Fig. 5g). In addition, mRNA levels of lipogenesis-related genes were upregulated in the liver tissues of HFD-fed mice and reversed by MLN4924 treatment (Fig. 5h). These results demonstrate that MLN4924 has preventive effect on HFD-induced hepatic steatosis.

### MLN4924 has therapeutic effect on the HFD-induced hepatic steatosis

**Fig. 6:**
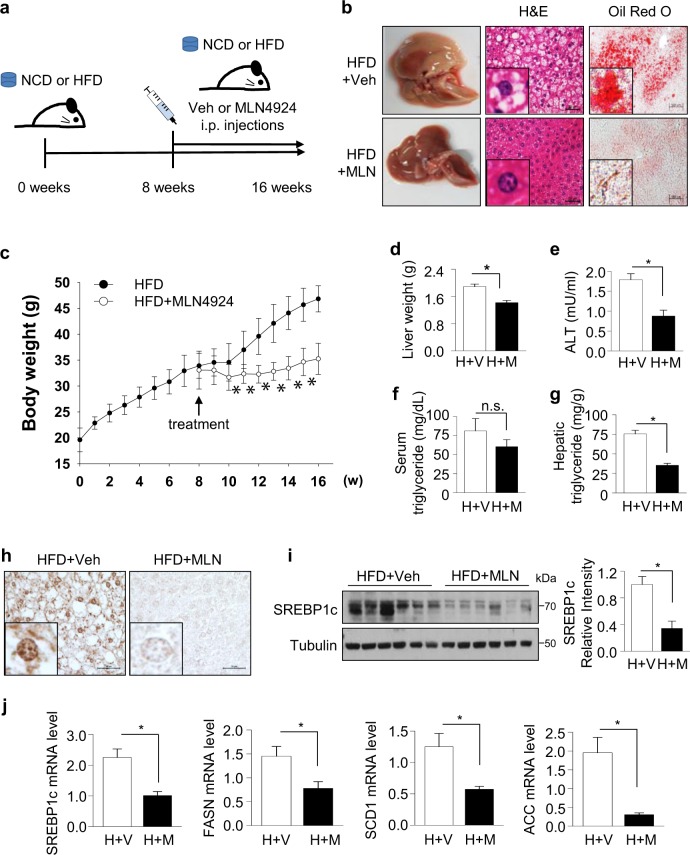
MLN4924 has therapeutic effect on the HFD-induced hepatic steatosis. **a**−**h** were fed the HFD for 8 weeks. After 8 weeks, the mice were divided into two groups, one fed HFD for 8 weeks and the other fed HFD and injected with MLN4924 (*n* = 6 per group). MLN4924 was injected intraperitoneally twice weekly for 8 weeks. **a** Schematic diagram of in vivo model. **b** Representative images showing the morphology, H&E staining, and Oil O Red staining of liver sections from HFD-fed mice (*n* = 6 images per group). **c** Mice were weighed for 16 weeks. **d** Liver weight was measured at the end of experiment (*n* = 6 per group). *P < 0.05 vs. control group. **e** ALT levels were measured in the serum of mice (*n* = 6 per group). **f** Triglyceride levels in the serum of mice (*n* = 6 per group). **g** Triglyceride concentrations in the liver tissue extracted from mice (*n* = 6 per group). **h** Immunohistochemical staining was applied to visualize the expression profile of SREBP1c in the liver of mice (*n* = 6 per group). **i** Expression level of SREBP1c in liver tissues of mice. The band intensities of SREBP1c protein were calculated using ImageJ and plotted by graph (mean ± SD, *n* = 6). **j** Total RNAs were extracted from liver tissues of mice. SREBP1c, ACC, FASN, and SCD1 mRNA levels were quantified by RT-qPCR. Results were quantified as the relative levels vs. 18S RNA level. All data were presented as the mean ± SD (*n* = 6 per group).

**Fig. 7:**
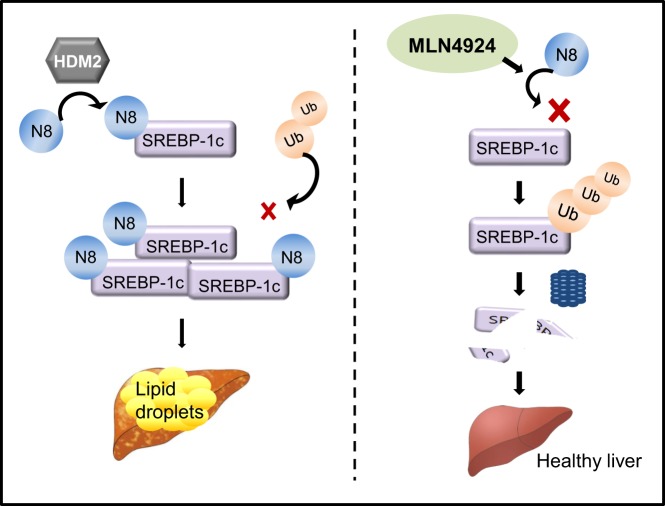
The proposed mechanism model. Neddylation of SREBP1c stabilizes SREBP1c protein through interfering with its ubiquitination, thus contributing to NAFLD progression. Conversely, MLN4924 suppresses NAFLD through blockade of SREBP1c neddylation and subsequent protein degradation.

Given the results that MLN4924 prevented HFD-induced overweight (Fig. 5c), to rule out the contribution of MLN4924 to obesity, we further examined whether MLN4924 has therapeutic effect on hepatic steatosis. As there was no significant difference between NCD mice and MLN4924-treated NCD mice, mice were fed with only HFD for 8 weeks, and then injected with MLN4924 or vehicle for a further 8 weeks (Fig. 6a). Upon histological examination, mice fed with HFD exhibited hepatic steatosis, while MLN4924-treated HFD mice showed a decrease in lipid droplet formation (Fig. 6b). Also, body and liver weight were also decreased in MLN4924-treated HFD-fed mice (Fig. 6c, d). However, there was no significant difference in food intake between vehicle and MLN4924-treated groups (Supplementary Fig. S1B). In addition, alanine aminotransferase (ALT) analysis indicated that MLN4924 prevented damage to liver from accumulating lipids (Fig. 6e). Although serum TG levels remained unchanged (Fig. 6f), hepatic TG levels were decreased in MLN4924-treated HFD-fed mice compared to those in HFD mice (Fig. 6g), suggesting that MLN4924 may not be associated with lipid catabolism or lipoprotein production. When the expressions of SREBP1c in liver tissues were confirmed by IHC and Western blotting, both showed a decrease of SREBP1c expression in MLN4924-treated mice (Fig. 6h, i). The induced expression of lipogenic genes in the liver tissues of HFD mice was also decreased by MLN4924 treatment (Fig. 6j). These results indicated that MLN4924 has a therapeutic effect on hepatic steatosis and it could be a novel agent for the treatment of NAFLD (Fig. 7).

## Discussion

In this study, we firstly investigated the hepatic role of SREBP1c neddylation during the development of hepatic steatosis, and found HDM2 targets SREBP1c for neddylation. Through blockade of SREBP1c ubiquitination, SREBP1c neddylation enhances its protein stability, and ultimately increases lipid accumulation in hepatocyte. We also found MLN4924, an E1 inhibitor of neddylation, shows preventive and therapeutic effects on hepatic steatosis. Our results clearly offer a new perspective for how neddylation enhances SREBP1c-dependent hepatic lipogenesis.

Hepatic lipogenesis, which is characteristic of NAFLD, is mainly regulated by the transcription factor SREBP1c^12,34^. It has been reported that SREBP1c is controlled by several molecular mechanisms. Nuclear SREBP1c is known to be controlled through the ubiquitin-proteasome pathway^35^. For instance, SREBP1c is phosphorylated at T426 and S430 residues in the HLH domain by GSK-3β, and consequently degraded by Fbw7-dependent ubiquitination^33^. We here found that the HLH domain of SREBP1c is the common site for neddylation and ubiquitination. Since the neddylation functionally competes with the ubiquitination of the domain, the high expression of NEDD8 upregulates the cellular level of SREBP1c in the liver. However, since the neddylated and ubiquitinated residues in SREBP1c are not identified so far, we do not know the precise mechanism underlying SREBP1c stabilization. This point remains to be investigated in the next study.

In the present study, we found that NEDD8 stabilizes SREBP1c in both the precursor and mature form. Since the cleaved SREBP1c alone is transcriptionally activated, the effect of NEDD8 on the cleavage of SREBP1c might be an important check-point for a better understanding of the SREBP1c-mediated steatosis, which is an open question to date. In addition, we in vitro and in vivo demonstrated that neddylation enhances the transcription of the SREBP1c gene. Given that SREBP1c is known to be induced by autoregulation^36^, the enhancement of SREBP1c transcription may be driven in a feedforward manner by the SREBP1c stabilized by NEDD8.

HDM2 was originally reported to bind to the tumor suppressor, p53, and inhibit p53-mediated transactivation by facilitating neddylation^37,38^ and function as an E3 ubiquitin ligase of p53, and thus downregulate protein stability and transcriptional activity^39^. We also previously reported that HDM2 neddylates PPAR-γ, and then promotes adipogenesis^21^. HDM2 also inhibits transcriptional activity of p73 through neddylation^40^. Given the role of HDM2 for inducing hepatic steatosis by decreasing p73 expression^41^, we hypothesized that HDM2 may possibly be involved in SREBP1c neddylation. As expected, HDM2 was shown to induce hepatic lipogenesis by exhibiting E3 ligase activity and then neddylating SREBP1c.

Currently, the most effective treatment for NAFLD involves lifestyle improvement such as dieting, exercise therapy and weight loss. In pharmacological therapy, UDCA has been studied as a potential therapeutic agent to treat hepatic steatosis by controlling the expression of SREBP1c^42^. UDCA has multiple hepatoprotective activities and is known to improve chronic liver diseases^7^. Nevertheless, the usage of UDCA is still a controversial issue in the treatment of hepatic steatosis due to its relative ineffectiveness in a clinical trial setting^8^. Besides UDCA, several drugs including insulin resistance modifiers, antioxidants and weight loss agents have been studied and used clinically for the treatment of hepatic steatosis; however, there are concerns about the safety of these drugs and the side effects associated with long-term use^10^. Given that only few effective drugs are available for the treatment of hepatic steatosis, trials to find more effective therapeutic agents are necessary for the clinical application.

MLN4924 is a specific small molecule inhibitor of the enzymatic action of NAE, mediated by MLN4924–NEDD8 adduct formation^22^. MLN4924 has been developed as an anticancer agent^43^ and passed the preclinical examination without any problems and is currently undergoing clinical tests^44^. A phase 1 study has been carried out on patients with metastatic melanoma. The 26 patients were treated with MLN4924 for 60-min intravenous infusions on 1-, 4-, 8- and 11-day cycles at various concentrations. No dose limiting toxicities (DLT) were seen when MLN4924 was injected at 89 or 209 mg/m^2^. For the 278 mg/m^2^ injection, patients experienced drug-related blood creatinine and bilirubin. However, it did not recur at the lower dose. So, the maximum tolerated dose was determined to be 209 mg/m^2^ because DLT showed no drug toxicity up to 209 mg/m^2 ^^23^. Since these results were based on anticancer effects, we used 1/3 lower concentrations to treat hepatic steatosis. As there was no difference of body weight, behavioral activity and pathological findings between vehicle and MLN4924 (30 mg/kg), the possibility of toxicity of the drug was ruled out.

Due to decrease of body weight in MLN4924-treated HFD mice (Fig. 5c) and given the previous results that MLN4924 effectively prevents the HFD-induced obesity^21^, it is difficult to determine whether MLN4924 directly inhibits NAFLD. To address this issue, we placed mice on HFD for 8 weeks, and administered MLN4924 for further 8 weeks. Although significant increase of body weight was observed in vehicle-treated HFD mice, that of MLN4924-treated HFD mice was slightly increased, suggesting that it is hard to rule out the effect of MLN4924 toward overall fat accumulation, even though it has a preventive effect on hepatic steatosis. Given its systemic effects, influences of NEDD8 or MLN4924 towards NAFLD should be evaluated using liver-specific NEDD8-deficient mice.

In conclusion, we suggest neddylation as a novel post-translation modification of SREBP1c during the progression of NAFLD. Given that NEDD8 is highly expressed in NAFLD patients and neddylation enhances SREBP1c-mediated hepatic lipogenesis through blockade of its ubiquitination, our results provide a clue to understand how SREBP1c contributes to the hepatic steatosis development. Moreover, we found preventive and therapeutic effects of MLN4924 on hepatic steatosis by reducing lipogenic gene expression and hepatic triglycerides through blockade of SREBP1c neddylation. Based on these results, we propose neddylation of SREBP1c as a new opportunity for the treatment of NAFLD and MLN4924 as a new therapeutic agent to treat hepatic steatosis.

## Materials and methods

### Animal models

This animal study was approved by the Seoul National University Animal Experiments Ethics Committee (approval #, SNU-150907-1-3). C57BL/6J mice (Central Lab. Animal Inc., Seoul, Korea) were housed in a pathogen-free facility under a 12-h light/12-h dark cycle. Four-week-old mice were fed a normal control diet (NCD) or HFD with 60% calories from fat (Research Diets Inc., New Brunswick, NJ) for 12 weeks. Vehicle or MLN4924 (30 mg/kg) was injected intraperitoneally twice a week. To assess the therapeutic effect of MLN4924 against hepatic steatosis, 4-week-old male C57BL/6J mice were fed HFD for 8 weeks. After 8 weeks, the mice were divided into two groups, and injected with vehicle or MLN4924 (30 mg/kg); they then continued with the HFD for the following 8 weeks. Food consumption was measured weekly for individually housed mice, and body weight was measured once a week. Liver tissues were rapidly excised after sacrifice at the end of the experimental period, weighed, and flash frozen in liquid nitrogen. Samples were stored at −80 °C and a portion of the liver was fixed in 4% formaldehyde.

### Human hepatic steatosis tissues

The clinical research component of this research was approved by the Institutional Review Board of Seoul National University Hospital (approval number C-1810-096-980). Liver tissues of ten hepatic steatosis patients and ten healthy subjects were collected at the tissue bank in Seoul National University Hospital. Detailed patient information is presented in Supplementary Table 1.

### Cell lines

Human embryonic kidney cells (HEK293) were obtained from the American Type Culture Collection (Manassas, VA). Human liver hepatocellular cells (HepG2) were obtained from the Korea Cell Bank (Seoul, Korea). HepG2 and HEK293 cells were cultured in Dulbecco’s modified Eagle’s medium supplemented with 10% fetal bovine serum and 1% penicillin/streptomycin.

### Plasmids, small interfering RNAs and transfection

PCR-amplified cDNAs of rat SREBP1c (403 amino acids, ~50 kDa) were inserted into Flag-tagged pcDNA3. FASN and ACRP30 luciferase vectors were described previously^45^. SREBP1c-TA (bp 1–36), SREBP1c-ZF (bp 146–206), and SREBP1c-HLH (bp 291–348) were amplified by PCR and inserted into Flag/SBP-tagged pcDNA3 (Clontech, Palo Alto, CA). His-NEDD8, a His-mutant NEDD8 (His-NEDD8ΔGG, which cannot be conjugated with the target protein), HA-Ubiquitin, Myc-SENP8, and pcHDM2 were constructed as described previously^21^. All small interfering RNAs (siRNAs) for each gene were synthesized by Integrated DNA Technologies (Coralville, IA). Plasmids and siRNAs were transiently transfected into cells using Lipofectamine 2000 (11668-019; Thermo Fisher Scientific, Waltham, MA) and Lipofectamine RNAiMAX (13778-150; Thermo Fisher Scientific) according to the manufacturer’s instructions. The sequences of siRNAs and plasmids are listed in Supplementary Tables 2 and 3.

### Identification of His-NEDD8 conjugates

Identification of NEDD8 conjugation was performed using Jaffray and Hay’s modified protocol^46^. After transfection of the His-NEDD8 or His-NEDD8ΔGG plasmid, cells were divided into two dishes. One set was lysed in 2× SDS sample buffer and analyzed by immunoblotting to check the expression level of proteins (input samples). The other was lysed by denaturing buffer (6 M guanidine hydrochloride, 0.1 M Na_2_HPO_4_/NaH_2_PO_4_, 0.01 M Tris-Cl (pH 8.0), 10 mM imidazole and 10 mM β-mercaptoethanol) for analysis of conjugates. The lysates were incubated with nickel beads for 4 h at room temperature. The beads were washed for 1 min in each step with the following solution: lysis buffer (pH 8.0), washing buffer 1 (pH 8.0, 8 M urea, 0.1 M Na_2_HPO_4_/NaH_2_PO_4_, 0.01 M Tris-HCl, 20 mM imidazole, 10 mM β-mercaptoethanol), washing buffer 2 (pH 6.3, 0.2% Triton X-100), and washing buffer 3 (pH 6.3, 0.1% Triton X-100). Finally, the beads were eluted using 2× SDS sample buffer and analyzed by Western blotting.

### mRNA expression profiling from public data

Hepatic gene expression analyses were conducted using the publicly available NCBI GEO dataset (www.ncbi.nlm.nih.gov/geo, GSE89632). Within the dataset, a healthy control group (*n* = 24) and a group of patients diagnosed with simple steatosis (*n* = 20) were selected and mRNA expression levels evaluated between the groups using the Mann–Whitney *U* test. For analyzing positive correlation coefficients within NEDD8 and hepatic steatosis-related genes, *r*^2^ value was calculated by Pearson correlation test.

### Quantitative RT-PCR

Total RNA samples from liver tissues and cultured cells were isolated using TRIzol Reagent (Invitrogen, CA, USA). cDNA synthesis and amplification were performed using an EasyScript cDNA Synthesis Kit (Applied Biological Materials Inc., Richmond, Canada). The cDNA of SREBP1c, ACC, SCD1, FASN, AGPAT and 18S rRNA was amplified with Evagreen qPCR master mix reagent (Applied Biological Materials) in StepOneTM Real-time PCR system (Applied Biosystems, CA, USA).

### Oil Red O staining

HepG2 cells were washed twice with phosphate buffered saline (PBS), fixed for 30 min with 3.7% formaldehyde in PBS and subsequently dehydrated with 60% isopropanol (MERCK, Darmstadt, Germany) for 5 min. After removing the isopropanol, 0.3% Oil Red O dye (O0625, Sigma-Aldrich, St. Louis, MO, USA) was added and incubated for 1 h. Then, the cells were thoroughly washed with 60% isopropanol until the background was clear. Frozen liver tissues (OCT compound-embedded, 20 μm) were fixed and stained with Oil Red O as described above. Images were obtained using the OLYMPUS IX71 microscope.

### Western blotting and immunoprecipitation analysis

Frozen liver tissues (60 mg) were lysed in ice-cold IP buffer supplemented with a cocktail of protease and phosphatase inhibitors (Sigma-Aldrich, St. Louis, MO, USA), and then centrifuged at 12,000 rpm for 10 min at 4 °C. Tissue extractions and cell lysates were separated on SDS-polyacrylamide gels and transferred to Immobilon-P membranes (Millipore, Billerica, MA, USA). Membranes were blocked with 5% skim milk in TBST (Tris-buffered saline containing 0.1% Tween 20) for 1 h, and incubated overnight with primary antibodies (dilution, 1:500‒1:3000) in the blocking solution. The membranes were further incubated with a horseradish peroxidase-conjugated secondary antibody for 1 h and visualized using the ECL Plus kit (Thermo Fisher Scientific, Waltham, MA, USA).

For immunoprecipitation assay, transfected cells were lysed with buffer containing 5 mM ethylenediaminetetraacetic acid (EDTA), 50 mM Tris-Cl, 100 mM NaCl, 0.1% NP-40, and a protease inhibitor cocktail (Sigma-Aldrich, St. Louis, MO, USA). Cell lysates (1.5 mg) were incubated with EZview red anti-flag M2 affinity beads (Sigma-Aldrich, St. Louis, MO, USA) for 16 h at 4 °C. For endogenous immunoprecipitation assay, cell lysates (1.5 mg) were incubated with anti-SREBP′-1c (10 μg) or anti-NEDD8 (10 μg) at 4 °C overnight and then with Protein A/G Sepharose beads^TM^ (GE Healthcare Life Sciences, Marlborough, MA, USA) for 4 h at 4 °C. After incubation, the precipitates were washed with lysis buffer three times. The proteins bound to the beads were eluted with 1× FLAG peptide (Sigma-Aldrich, St. Louis, MO, USA) or 2× SDS denaturing buffer and subjected to Western blotting. The antibodies used for Western blotting are listed in Supplementary Table 4.

### Reporter assay

Luciferase reporter genes, containing sterol regulatory element of the fatty acid synthase (FASN*)* gene and the adiponectin (ACRP30) gene, were a kind gift from Dr. Prof. Jae Bum Kim (Seoul National University, Seoul, South Korea). HEK293 cells were cotransfected with 1 μg each of reporter gene, Myc-SREBP1c, His-NEDD8 and β-gal plasmid. After stabilization for 48 h, luciferase activities were measured using a Lumat LB9507 luminometer (Berthold Technologies, Bad Wildbad, Germany) and normalized to β-gal activities. HepG2 cells were cotransfected with 1 μg each of reporter gene, Myc-SREBP1c and β-gal plasmid. After stabilization for 24 h, cells were treated with MLN4924 for 24 h. Luciferase activities were measured as described above.

### Nuclear extraction

HepG2 cells were immediately washed twice with ice-cold PBS (pH 7.8). Cells were scraped off the dishes, and the cell pellets were obtained by centrifugation (3000 rpm, 5 min, 4 °C). Then pellets were lysed at 4 °C with 0.3 ml buffer A (20 mM Tris-Cl (pH 7.8), 10 mM KCl, 1.5 mM MgCl_2_, 0.2 mM EDTA, 0.5 mM dithiothreitol, 0.5 mM phenylmethylsulfonyl fluoride (PMSF), 1 mM Na_3_VO_4_ and protease inhibitor cocktail). After 10 min on ice, cells were further lysed by the addition of 0.6% NP-40, and centrifuged (6000 rpm, 5 min, 4 °C) to obtain the cytosolic supernatant. The supernatant was stored at −20 °C (cytosolic extract) and the pellets were re-suspended in ice-cold nuclear extraction buffer (buffer A + 5% glycerol, 400 mM NaCl). Samples were incubated on ice for 30 min and centrifuged (12,000 rpm, 10 min, 4 °C) to obtain the nuclear supernatant. Cytosolic and nuclear proteins were subjected to Western blotting.

### Liver histology and immunohistochemistry

Excised livers were fixed with 4% paraformaldehyde and embedded in paraffin. Sections (4 μm) were routinely stained with hematoxylin and eosin (H&E). For immunohistochemistry (IHC), serial sections (4 μm) were cut from each paraffin block, deparaffinized, rehydrated in a graded alcohol series, heated in 10 mM sodium citrate (pH 6.0) using a microwave for 7 min to retrieve antigens. The sections were blocked with blocking solution (PBS containing 2.5% bovine serum albumin, 0.2% Triton X-100 and 10% normal goat serum) for 1 h, and incubated overnight at 4 °C with SREBP1c antibodies. Then the slides were incubated with horseradish peroxide (HRP) polymer at room temperature for 1 h, and the sections were visualized with ABC kit (VECTOR Laboratories LTD, Burlingame, CA) and DAB kit (DAKO, Denmark).

### Biochemical assays

Serum alanine aminotransferase (ALT) levels were determined by using Alanine Aminotransferase Activity Colorimetric/Fluorometric Assay Kit (Biovision, Milpitas, CA, USA), according to the manufacturer’s instruction. Serum TG and liver TG levels were measured by using an EnzyChrom™ Triglyceride Assay Kit (Bioassay Systems, Hayward, CA, USA), according to the manufacturer’s instruction.

### Statistical analysis

Means and standard deviation (SD) and standard error of the mean (SEM) were calculated using Microsoft Excel 2010 or Graph Pad Prism 5 software. The means of the two groups were compared using two-tailed and unpaired Student’s *t* test. The difference was considered statistically significant when *P < 0.05 versus control group.

## Supplementary information

Supplemental Table S1

Supplemental Table S2

Supplemental Table S3

Supplemental Table S4

Supplemental Figure S1

Supplemental information
