# Supplementary material for: Neddylation of sterol regulatory element-binding protein 1c is a potential therapeutic target for nonalcoholic fatty liver treatment
Source: Cell Death Dis. 2020 Apr 24;11(4):283. doi: 10.1038/s41419-020-2472-6 (PMC7181738; doi:10.1038/s41419-020-2472-6)
Supplement: Supplementary file 1 — Supplemental Table S1 [file 41419_2020_2472_MOESM1_ESM.docx]

**Supplemental Table S1** Patient information for hepatic steatosis

| **Patient No.** | **Diagnosis** | **Bxmacro(Rt)** | **Bxmicor(Rt)** |
| --- | --- | --- | --- |
| 1 | Healthy | 0.0 | 0.0 |
| 2 | Healthy | 1.0 | 1.0 |
| 3 | Healthy | 1.0 | 1.0 |
| 4 | Healthy | 0.0 | 0.0 |
| 5 | Healthy | 0.0 | 0.0 |
| 6 | Hepatic steatosis | 10.0 | 5.0 |
| 7 | Hepatic steatosis | 10.0 | 20.0-30.0 |
| 8 | Hepatic steatosis | 30.0 | 30.0 |
| 9 | Hepatic steatosis | 30.0 | 10.0 |
| 10 | Hepatic steatosis | 10.0 | 10.0 |
| 11 | Healthy | 0.0 | 0.0 |
| 12 | Healthy | 0.0 | 0.0 |
| 13 | Healthy | 0.0 | 0.0 |
| 14 | Healthy | 0.0 | 0.0 |
| 15 | Healthy | 1.0 | 1.0 |
| 16 | Hepatic steatosis | 10.0 | 10.0 |
| 17 | Hepatic steatosis | 10.0 | 10.0 |
| 18 | Hepatic steatosis | 15.0 | 15.0 |
| 19 | Hepatic steatosis | 20.0 | 20.0 |
| 20 | Hepatic steatosis | 15.0 | 15.0-20.0 |
