# Supplementary material for: Neddylation of sterol regulatory element-binding protein 1c is a potential therapeutic target for nonalcoholic fatty liver treatment
Source: Cell Death Dis. 2020 Apr 24;11(4):283. doi: 10.1038/s41419-020-2472-6 (PMC7181738; doi:10.1038/s41419-020-2472-6)
Supplement: Supplementary file 2 — Supplemental Table S2 [file 41419_2020_2472_MOESM2_ESM.docx]

**Supplemental Table S2** Sequence of siRNAs for knock down experiments

|  | **GenBank accession number** | **Sequencing5'---3'** |
| --- | --- | --- |
| Human NEDD8-I | NM_006156 | AGCGGUAGGAGCAGCAAUUUAUCCG |
| Human NEDD8-II | NM_006156 | GAAGAUGCUAAUUAAAGUGAAGACG |
| Human APPBP1-I | NM_003905 | GGACAAUCCAGAUAAUGAAAUAGTG |
| Human APPBP1-II | NM_003905 | GGAUCUACGACUAGAUAAGCCAUTT |
| Human HDM2- I | NM_002392 | UUCCUGAAGCUCUUGUACAAGGUCCUU |
| Human HDM2-II | NM_002392 | GCAAUGAUCUACAGAAAUUUAGUGG |
| Non-target |  | UUGAGCAAUUCACGUUCAUTT |
