# Supplementary material for: Neddylation of sterol regulatory element-binding protein 1c is a potential therapeutic target for nonalcoholic fatty liver treatment
Source: Cell Death Dis. 2020 Apr 24;11(4):283. doi: 10.1038/s41419-020-2472-6 (PMC7181738; doi:10.1038/s41419-020-2472-6)
Supplement: Supplementary file 3 — Supplemental Table S3 [file 41419_2020_2472_MOESM3_ESM.docx]

**Supplemental Table S3** Primers for Real-time PCR detection

| **Gene** |  | **Sequence5'---3'** |
| --- | --- | --- |
| Mouse SREBP1c | F | GGAGACCATGGATTGCACATT |
|  | R | GGAAGTCACTGTCTTGGTTGTTGA |
| Mouse FASN | F | AAGTTGCCCGAGTCAGAGAA |
|  | R | CGTCGAACTTGGAGAGATCC |
| Mouse ACC | F | TGAATCTCACGCGCCTACTATG |
|  | R | ATGACCCTGTTGCCTCCAAAC |
| Mouse SCD1 | F | GCGATACACTCTGGTGCTCA |
|  | R | CCCAGGGAAACCAGGATATT |
| Mouse AGPAT | F | CTGCTGCTCCACGTCAAATA |
|  | R | AGCCAGCCCATAGTAGCTCA |
| Human ACC | F | GTTGCACAAAAGGATTTCAG |
|  | R | CGCATTACCATGCTCCGCAC |
| Human FASN | F | ACAGGGACAACCTGGAGTTCT |
|  | R | CTGTGGTCCCACTTGATGAGT |
| 18S | F | TTCGTATTGAGCCGCTAGA |
|  | R | CTTTCGCTCTGGTCCGTCTT |
