# Supplementary material for: Neddylation of sterol regulatory element-binding protein 1c is a potential therapeutic target for nonalcoholic fatty liver treatment
Source: Cell Death Dis. 2020 Apr 24;11(4):283. doi: 10.1038/s41419-020-2472-6 (PMC7181738; doi:10.1038/s41419-020-2472-6)
Supplement: Supplementary file 4 — Supplemental Table S4 [file 41419_2020_2472_MOESM4_ESM.docx]

**Supplemental Table S4** List of antibodies for western blot

| **Antibody** | **Cat No.** | **Manufacturer** | **Species** |
| --- | --- | --- | --- |
| HDM2 | sc-965 | Santa Cruz | mouse |
| β-tubulin | sc-9104 | Santa Cruz | rabbit |
| Ubiquitin | sc-9133 | Santa Cruz | rabbit |
| Lamin B | sc-374015 | Santa Cruz | mouse |
| NEDD8 | 2735 | Cell signaling | rabbit |
| Myc | 2278 | Cell signaling | rabbit |
| APPBP1 | NBP1-92162 | NOVUS | rabbit |
| SREBP-1c | 557036 | BD Biosciences | mouse |
| Anti-HA | 11867423001 | Roche | Rat |
| Anti-flag M2 | F3165 | Sigma-Aldrich | mouse |
| LXR alpha | Ab28478 | Abcam | rabbit |
