# Supplementary figures and images for: Neddylation of sterol regulatory element-binding protein 1c is a potential therapeutic target for nonalcoholic fatty liver treatment
Source: Cell Death Dis. 2020 Apr 24;11(4):283. doi: 10.1038/s41419-020-2472-6 (PMC7181738; doi:10.1038/s41419-020-2472-6)

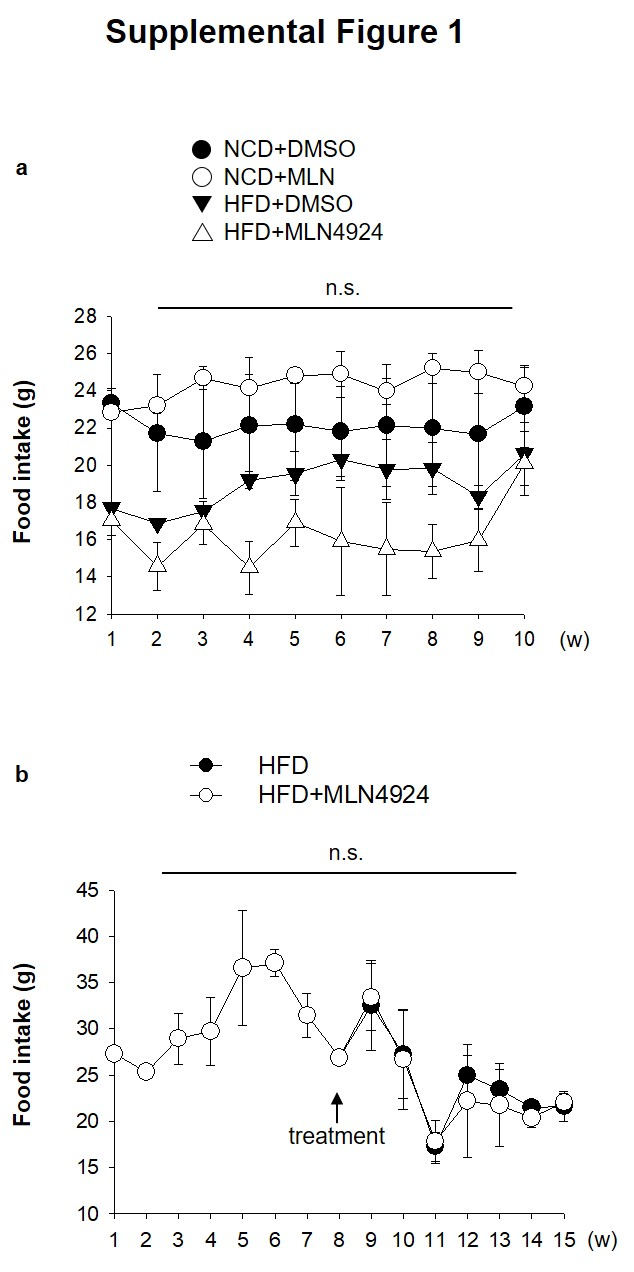

Supplement: Supplementary file 5 — Supplemental Figure S1 [file 41419_2020_2472_MOESM5_ESM.tif]
