# Supplementary material for: Neddylation of sterol regulatory element-binding protein 1c is a potential therapeutic target for nonalcoholic fatty liver treatment
Source: Cell Death Dis. 2020 Apr 24;11(4):283. doi: 10.1038/s41419-020-2472-6 (PMC7181738; doi:10.1038/s41419-020-2472-6)
Supplement: Supplementary file 6 — Supplemental information [file 41419_2020_2472_MOESM6_ESM.docx]

**Supplemental Table legends**

**Supplemental Table S1** Patient information for hepatic steatosis

**Supplemental Table S2** Sequence of siRNAs for knock down experiments

**Supplemental Table S3** Primers for Real-time PCR detection

**Supplemental Table S4** List of antibodies for western blot

**Supplemental Figure legends**

**Supplemental Figure S1 No significant difference was observed in the amount of food intake between mice groups**

(A) Mice were divided into four groups (*n* = 9 per group): vehicle treatment of NCD-fed mice or HFD-fed mice, MLN4924 treatment (30 mg/kg) of NCD-fed mice or HFD-fed mice. Mice were fed HFD for 12 weeks, and MLN4924 was injected intraperitoneally twice weekly for 12 weeks. Food consumption was measured once a week. (B) were fed the HFD for 8 weeks. After 8 weeks, the mice were divided into two groups, one fed HFD for 8 weeks and the other fed HFD and injected with MLN4924 (*n* = 6 per group). MLN4924 was injected intraperitoneally twice weekly for 8 weeks. Food consumption was measured weekly.
